# Obituary

**Published:** 1894-07

**Authors:** 


### Obituary.

DR. HENRY VAN AERNAM, of Franklinville, N. Y., died at his residence in that village on Friday, June 1, 1894, at the age of seventy-five years.

Dr. Van Aernam was, for more than a third of a century, a man of prominence, and had lived in Franklinville for forty-six years.

From the very earliest period of his professional career he took a prominent place in medicine and enjoyed a large practice. Prior to the outbreak of the civil war he served one term in the State assembly, declining a reëlection on account of his professional engagements. When the war came, General Patrick H. Jones raised the 154th regiment in the Southern tier and Dr. Van Aernam offered his services as surgeon, which were promptly accepted by Governor Morgan. The military authorities were not slow to recognize Dr. Van Aernam's ability as a surgeon, as well as his capacity as an organizer, hence he moved rapidly forward from regimental to brigade and division duty, in all of which he served faithfully and meritoriously. The writer met him in the woods of Chancellorsville on the night of May 2, 1863, after the 11th corps had been driven in on the Union right and was in utter rout; but Dr. Van Aernam was then, as always, calm, self-possessed, hopeful and even aggressive. Later the 11th army corps was sent to the West and Dr. Van Aernam performed his final military duty when Sherman marched to the sea and thence through the Carolinas to Bentonville. He was elected to the Thirty-ninth Congress as a republican and was reëlected to the Fortieth Congress, serving with conspicuous prominence during the reconstruction period. General Grant, upon his inauguration as president, appointed Dr. Van Aernam as commissioner of pensions, and during his administration of the office many improvements were instituted and the basis of all that is good in the present system was developed. It is not saying too much to assert that Dr. Van Aernam was the best commissioner of pensions who has ever held that office. In 1878 he was again sent to Congress only to be reëlected two years later.

A few years ago he had a paralytic stroke and has been in feeble health since that time. Dr. Van Aernam was a power in politics, always standing for everything that was pure and of the better sort, while in medicine he was easily the most conspicuous physician in his region of the country. His advice was often sought by patients from a great distance and his consulting practice was a large one. His door was always open to his professional, political and soldier friends, and they often flocked to his house in great numbers to pay their respects or to consult with him on personal or public affairs.

Dr. Van Aernam was married nearly fifty years ago, his wife and two children surviving him. His son, Charles D. Van Aernam, is the present supervisor of Franklinville, and his daughter is the widow of the late surrogate, Hon. James D. McVey. His funeral was held on Monday, June 4th, and was largely attended by veteran soldiers, civilians, men in public life, physicians and friends. The bearers were A. P. Adams, Dr. H. D. Walker, Wm. Ely, Dr. R. Terry, Wm. Swinton and Dexter C. Weed, all old and warm friends of the deceased.

As a mark of respect, nearly all the business places in Franklinville were closed during the funeral, as was also the first room of the district school. Ten Broeck Academy, of which Dr. Van Aernam was one of the honored trustees, was kept closed all day.

He was buried in the family lot in beautiful Mount Prospect cemetery, which is located on a hill overlooking the fine little village he was pleased to call his home. Messages and letters of sympathy and condolence were received by the family by telegraph and post from his former colleagues in public life, and from warm personal friends residing in all quarters of the United States.

### RESOLUTIONS OF RESPECT.

At a meeting of the physicians of Cattaraugus county, held in Franklinville on June 4th, Dr. E. S. Stewart, of Ellicottville, chairman, and Dr. H. D. Walker, of Franklinville, secretary, the following resolutions were unanimously adopted :

WHEREAS, Death having removed from among us our esteemed friend and fellow-physician, Dr. Henry Van Aernam, we, here assembled, desire to express our high appreciation of his worth as a citizen, a friend and a physician.

RESOLVED, That in his death our profession has sustained the loss of an honest and skilful physician and surgeon, whose counsel we shall hereafter greatly miss.

RESOLVED, That we extend to his family our heartfelt sympathy in this their sad bereavement.

RESOLVED, That a copy of these resolutions be sent to the family of the deceased, to the BUFFALO MEDICAL AND SURGICAL JOURNAL and the local and county papers.

Signed, Drs. E. S. Stewart, C. H. Bartlett, J. L. Eddy, J. P. Colgrove, C. D. McLouth, F. Findlay, Ranson Terry, H. D. Walker and J. W. Kales.

---

DR. ELIJAH S. ELDER, of Indianapolis, Ind., died May 9, 1894, of peritonitis following intestinal obstruction, aged fifty-three years. Dr. Elder was president of the Indiana State Medical Society, Dean and Professor of Principles and Practice of Medicine in the

Medical College of Indiana and had been for many years connected with the *Indiana Medical Journal.* Dr. Elder was a man of prominence in the medical profession, of great integrity of character and a physician who will be greatly missed not only in the city of his home, but by a large circle of friends and acquaintances widely scattered throughout the country.

